# Characteristics of hypoglycaemic episodes in diabetic patients treated at the emergency room of a Portuguese tertiary centre, 2012‐2016

**DOI:** 10.1002/edm2.150

**Published:** 2020-05-24

**Authors:** Catarina A. Pereira, Raquel Almeida, Jorge Dores

**Affiliations:** ^1^ Endocrinology Department Hospital University Centre of Porto Porto Portugal

**Keywords:** diabetes mellitus, emergency room, hypoglycaemia

## Abstract

**Objective:**

To analyse changes in the characteristics of hypoglycaemic episodes treated in the emergency room of a tertiary hospital in Portugal between 2012 and 2016.

**Research Design and Methods:**

We retrospectively analysed all emergency room reports for patients discharged with a diagnosis of hypoglycaemia between 2012 and 2016 and analysed demographic characteristics, type of diabetes and treatments, causes of hypoglycaemia and discharge destination. Patients without diabetes were excluded.

**Results:**

In total, 676 hypoglycaemic episodes were analysed. Most patients were female (59%) and the median age of the patients was 71 years (interquartile range, 57‐81). The proportion of hypoglycaemic episodes relative to all emergency episodes decreased from 1.5% in 2012 to 1.0% in 2016 (*P* < .001). The proportion of patients with type 1 diabetes increased from 15.6% to 23.8%, while that of patients with type 2 diabetes decreased from 80.3% to 72.3% (nonsignificant differences). There was an increase in the use of insulin (67.1% to 85.4%, *P* = .02) and a decrease in the use of insulin secretagogues (26.6% to 11.5%, *P* = .03) over the study period. The rate of hospitalization dropped significantly from 11% in 2012 to 4.3% in 2015 and 5.4% in 2016 (*P* = .02).

**Conclusions:**

Despite the increasing use of newer diabetes medications associated with a lower risk of hypoglycaemia, these episodes still require emergency care. The proportion of patients receiving insulin increased over the years, probably due to the slight increase in the prevalence of type 1 diabetes and the increasing replacement of secretagogues with insulin in type 2 diabetes.

## Graphical abstract

We present the changes in the characteristics of hypoglycaemic episodes treated in the emergency room of a tertiary hospital in Portugal between 2012 and 2016. With the analysis of 676 hypoglycaemic episodes, we can better understand and reflect on the reasons why, despite the increasing use of newer diabetes medications associated with a lower risk of hypoglycaemia, these episodes still require emergency care.

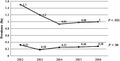

## 1 INTRODUCTION

Diabetes is a growing health concern. In 2017, an estimated 425 million people globally had diabetes and this figure is expected to reach 629 million by 2045.^1^ Although many treatments exist for diabetes, just over 50% of patients with type 2 diabetes achieve the American Diabetes Association glycated haemoglobin (HbA1c) goal of <7.0% (53 mmol/mol).^2^ Concern about hypoglycaemia is a barrier to optimal diabetes care,^3^ and severe hypoglycaemia is the most serious adverse effect of insulin therapy in patients with type 1 diabetes.^4^, ^5^

Hypoglycaemia can cause acute harm to patients and others, especially when it causes car accidents, falls or other injuries.^6^ Other severe consequences include vision loss, neurocognitive dysfunction, cerebrovascular disease and myocardial infarction.^7^ Increased cardiovascular risk has also been reported, with an increased incidence of cardiac arrhythmias during acute hypoglycaemia^8^ and an increased risk of thrombosis.^9^ Hypoglycaemic episodes have also been linked to lower treatment satisfaction and adherence.^10^ In the older population, severe hypoglycaemia is associated with a greater risk of dementia ^11^ and death.^12^, ^13^, ^14^ Finally, hypoglycaemia poses a significant economic burden on both society and healthcare systems.^15^

Hypoglycaemia is a common reason for emergency room (ER) visits and hospitalization. Between 1999 and 2011, over 400,000 people were admitted to US hospitals with hypoglycaemia, outnumbering those admitted for hyperglycaemia.^16^ Based on data for 2013‐2014, hypoglycaemic episodes accounted for 13.3% of all ER visits for adverse drug events in the United States.^17^ In Portugal, the HIPOS‐ER (Hypoglycemia in Portugal Observational Study–Emergency Room) study showed that 0.074% of type 2 diabetic patients required emergency care for severe hypoglycaemia.^18^

The aim of this study was to describe the characteristics of hypoglycaemic episodes in diabetic patients discharged from a Portuguese ER with a diagnosis of hypoglycaemia between 2012 and 2016.

## 2 RESEARCH DESIGN AND METHODS

We performed a retrospective observational study of all diabetes‐related episodes of hypoglycaemia treated at the ER of a tertiary hospital in Portugal between 2012 and 2016. We reviewed all ER reports for this period containing the following discharge ICD‐9 (International Classification of Diseases, Ninth Revision) codes: ‘specified hypoglycaemia’, ‘nonspecified hypoglycaemia’ and ‘hypoglycaemic coma’. Confirmed hypoglycaemic events only (capillary or venous plasma glucose level <70 mg/dL) were considered. ER admissions for nondiabetic hypoglycaemia were analysed separately from the episodes in people with diabetes. Admissions in which the report did not specify whether the patient had diabetes or not were excluded.

We recorded demographic characteristics, dependence on others, type of diabetes, treatments, levels of consciousness on admission, causes of hypoglycaemia, and destination at discharge from the ER for all patients included and compared data over the 5 years analysed.

Statistical analysis was performed in SPSS version 23. Chi‐square, Mann‐Whitney and Kruskal‐Wallis tests were used. Results are presented as median (interquartile range [IQR]).

## 3 RESULTS

A total of 676 hypoglycaemic episodes (597 patients) treated at the ER of our hospital between 2012 and 2016 were analysed. The episodes occurred in 399 (59%) females and 277 males with a median age of 71 years (IQR, 57‐81). The breakdown of episodes and demographic information per year is presented in Table 1. The percentages were calculated on the basis of the information available.

**Table 1:** Number of annual emergency room visits and episodes of diabetic and nondiabetic hypoglycaemia between 2012 and 2016 and characteristics of patients with diabetes‐related hypoglycaemia

	2012	2013	2014	2015	2016
Total emergency room episodes	117 231	118 750	122 049	119 578	126 710
Hypoglycaemic episodes (diabetes + nondiabetes)	211	181	150	158	178
Hypoglycaemic episodes (diabetes)	173	142	114	117	130
Female (%)	105 (60.7%)	92 (64.8%)	68 (57.9%)	65 (55.6%)	69 (53.1%)
Median age (P25‐P75)	72 (58‐82)	70 (58‐82)	73 (60‐81)	70 (55‐79)	67 (56‐81)

**Figure 1:**
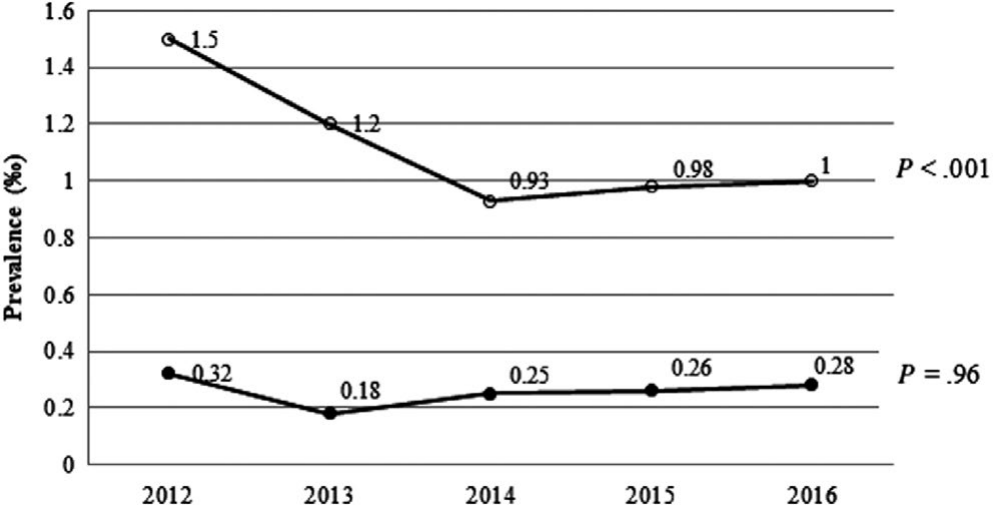
Changes in proportion of diabetic and nondiabetic hypoglycaemic episodes relative to total emergency room episodes between 2012 and 2016. White circles = Diabetes; Black circles = Nondiabetes

Hypoglycaemic episodes as a proportion of all ER admissions fell from 1.5‰ in 2012 to 1.0‰ in 2016 (*P* < .001) in patients with diabetes and remained relatively stable in patients without diabetes (Figure 1). Most patients (77.9%; 511/656) had type 2 diabetes. We observed an increase in ER admissions for hypoglycaemia in patients with type 1 diabetes (from 15.9% (27/170) in 2012 to 24.0% (31/129) in 2016, *P* = .42) and a decrease in patients with type 2 diabetes: 81.8% (139/170) in 2012 to 72.9% (94/129) in 2016; *P* = .44. These changes, however, were not statistically significant.

**Figure 2:**
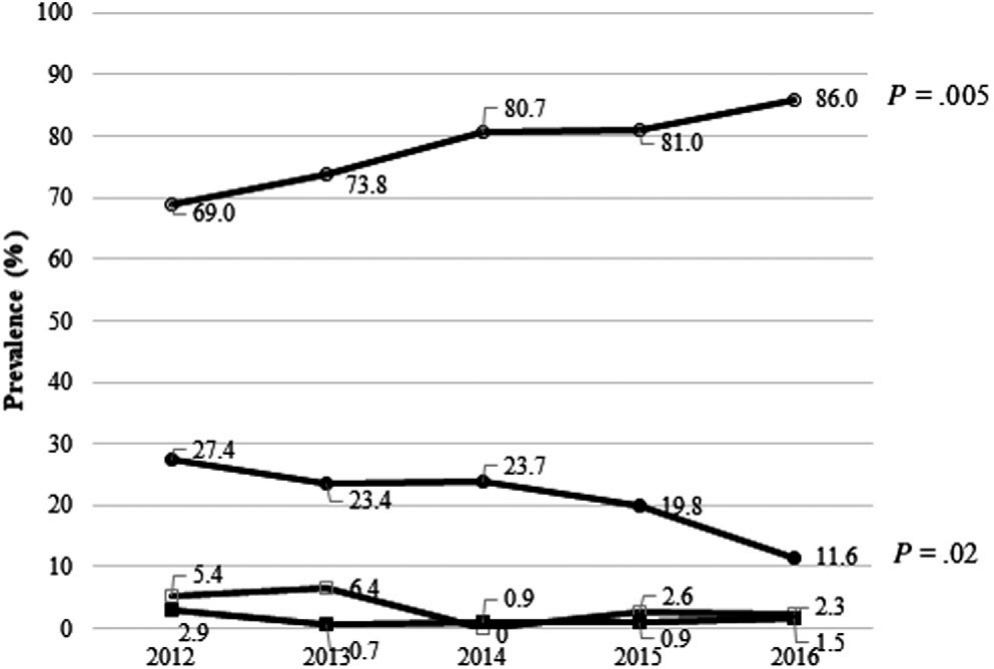
Changes in prevalence of type of antidiabetic treatment in patients discharged from the emergency room with a diagnosis of hypoglycaemia. White circles = Insulin; Black circles = Sulphonylureas; White squares = Nonsecretagogues; Black squares = Unknown

Insulin therapy was the most common treatment, and its prevalence increased steadily from 69.0% (116/168) in 2012 to 86.0% (111/129) in 2016 (*P* = .005). By contrast, we observed a persistent decrease in the proportion of patients being treated with sulphonylureas: from 27.4% (46/168) in 2012 to 11.6% (15/129) in 2016; *P* = .02 (Figure 2). Sixteen patients (2.4%) were treated with concomitant sulphonylurea and insulin. The proportion of patients exclusively treated with insulin analogues increased steadily from 36.4% (59/162) in 2012 to 55.7% (59/106) in 2016; *P* = .01, while that of patients on NPH insulin therapy (including biphasic human insulin) fell from 29.0% (47/162) in 2012 to 19.8% (21/106) in 2016. The difference in this second case was not significant (*P* = .18). The prevalence of intensive therapy with insulin analogues (combination of basal and prandial insulin) increased from 9.3% (15/162) in 2012 to 22.3% (23/103) in 2015 and dropped slightly to 20.8% (22/106) in 2016 (*P* = .03). Eight patients with an insulin pump were included in this group.

Almost one‐third of patients (31.4%; 204/650) were dependent to some degree on other people for performing daily activities. This rate did not change significantly over the years analysed, although we can observe a slight reduction (from 34.5% to 28.5%; *P* = .28). In 31.9% (199/624) of cases, a close family member was responsible for administering the diabetes medication.

The vast majority of patients were conscious when admitted to the ER; only 6.5% (43/664) had a Glasgow Coma Score under 13. Neuroglycopenic clinical signs were reported for approximately 73% (409/563) of patients.

Cause of hypoglycaemia was mentioned in 512 (75.7%) of the 676 ER reports. The main causes were reduced food intake (40.4%; 204/512,), overtreatment (33.6%; 172/512) and drug misuse (12.7%; 65/512) (Table 2).

**Table 2:** Causes for hypoglycaemia

Reduced food intake/altered dietary timing	207
Overtreatment	172
Drug misuse/error	65
Acute kidney injury	17
Unusual physical activity	13
Alcohol consumption	11
Nonadherence to treatment regimen	7
Other causes	20
Not reported	164

Eighty‐seven per cent of patients (588/676) were discharged home, while 8.1% (55/676) were admitted to hospital. The most common reason for admission was medication with sulphonylurea with prolonged time of action (15/55), followed by decompensated chronic illness (7/55), acute renal failure (7/55) and pneumonia (7/55). Admission rates decreased significantly over the years: from 11% (19/173) in 2012 to 4.3% (5/117) in 2015 and 5.4% (7/130) in 2016, *P* = .02. Median length of stay in the ER was 8 hours (IQR, 5‐14.5) and was longer for patients taking sulphonylureas than for those taking insulin (12 vs 8 hours, *P* < .001). Hospitalization was also significantly more common in patients on sulphonylureas: 22.7% (29/128) vs. 4.2% (21/496) for patients taking insulin; *P* < .001. Patients taking both sulphonylurea and insulin were excluded from this analysis.

## 4 CONCLUSIONS

The proportion of ER visits for diabetic patients discharged with a diagnosis of hypoglycaemia at our hospital decreased from 2012 to 2016. Nevertheless, Lipska et al found an increasing trend in admissions for hypoglycaemia in a national US survey in Medicare beneficiaries performed between 1999 and 2011.^16^ This can be attributed to the greater proportion of patients achieving the recommended thresholds for HbA1c,^2^, ^19^ given the increasing awareness in recent years about the benefits of good glycaemic control in the prevention of chronic diabetes complications.

The median age of the patients in our series, 71 years (IQ, 57‐81), is the same as that reported by Su et al^20^ in a study of predisposing factors for hypoglycaemia in the ER but lower than that of 77.5 years reported in the HIPOS‐ER study.^18^ Su et al^20^ found an almost equal distribution of men and women in their series, whereas both our study and the HIPOS‐ER^18^ study found that slightly more women than men required emergency treatment for hypoglycaemia (59% and 57.6%, respectively).

Over 70% of diabetes‐related hypoglycaemia events treated each year by the ER at our hospital occurred in patients with type 2 diabetes. Apart from their far higher numbers, patients with type 2 diabetes are more likely to require emergency care because they are generally older and frailer than patients with type 1 diabetes and are also more likely to have a higher burden of comorbidity and polypharmacy. Nevertheless, we did observe a decrease in the proportion of patients with type 2 diabetes presenting with hypoglycaemia requiring emergency care over the study period, probably because of the increasing use of antidiabetic drugs other than sulphonylureas. This hypothesis is supported by the steady decrease observed in ER patients with hypoglycaemia on sulphonylureas.

Conversely, we found an increase in the proportion of patients with type 1 diabetes admitted to the ER for hypoglycaemia. This is probably linked to the decrease observed for patients with type 2 diabetes and the higher prevalence of patients with type 1 diabetes under tight control with intensive insulin therapy.

The decrease in the proportion of patients taking sulfonylureas and the increase in those on insulin therapy have two likely explanations: the slight increase observed in hypoglycaemia among patients with type 1 diabetes and the increasing proportion of patients with type 2 diabetes being treated with insulin rather than insulin secretagogues. We also observed a decrease in the proportion of patients on NPH insulin and an increase in the use of insulin analogues, mainly as part of an intensive therapy regimen. It is well known that insulin analogues are associated with fewer hypoglycaemic events than traditional human insulins in both type 1 and type 2 diabetes.^21^, ^22^, ^23^, ^24^ Our results are therefore probably indicative of the increasing use of insulin analogues than of an increased risk of hypoglycaemia during this treatment.

In a survey of treatment‐related severe hypoglycaemia in Japanese patients, Namba et al ^25^ reported causes of hypoglycaemia similar to our study: inappropriate diet or dietary timing, excessive alcohol intake and human errors with drug misuse. In about one‐third of the patients in our series, the only cause of hypoglycaemia identified was overdose of insulin or sulphonylurea, which was corrected in the ER.

The overall hospitalization rate of 8.7% detected in our series for diabetic patients with a diagnosis of hypoglycaemia at discharge from the ER is much lower than the rates of 25%^26^ and 29.3%^27^ reported for ER visits in the United States and than the rate of hospitalizations reported on HIPOS‐ER (44.1%).^18^ Moreover, the annual hospitalization rates decreased steadily from 11% in 2012 to 4.3% in 2015, with a slight peak in 2016 (5.4%). We believe that this decrease can be explained by the lower use of sulphonylureas, especially long‐acting, in the later years, as hypoglycaemic patients on these drugs were more likely to be hospitalized in both our series and the HIPOS‐ER series.^18^

As ours is a retrospective study, the conclusions that can be drawn are limited. One limitation is that the cases of hypoglycaemia were identified solely on the basis of discharge ICD‐9 codes mentioned in the ER reports. We can speculate that these diagnoses are underestimated, as in our experience patients admitted for hypoglycaemia may be discharged with a diagnosis of a complication or an underlying disease that caused hypoglycaemia (eg trauma or aspiration pneumonia and acute kidney injury) rather than hypoglycaemia. This can also explain our much lower rate of hospitalization comparing with HIPOS‐ER.^18^

In conclusion, our findings show that ER visits for hypoglycaemia are dropping, probably due to the growing use of new antidiabetic drugs to replace sulphonylureas in patients with type 2 diabetes. Accordingly, more and more patients admitted to the ER for hypoglycaemia are on insulin therapy. We also detected an increasing use of insulin analogues compared with human insulins, but cannot confirm whether this change contributed somehow to the decrease in hypoglycaemic episodes seen at our ER. The advanced age of our patients did not vary significantly over the years studied, although we did observe a slight reduction in the proportion of frail patients and patients dependent on other people to administer their diabetes medication.

## CONFLICT OF INTEREST

The authors have no conflicts of interest to disclose.

## AUTHORS' CONTRIBUTIONS

Catarina A. Pereira wrote the manuscript, researched and analysed data. Raquel Almeida researched data. Jorge Dores designed the study, contributed to the discussion and reviewed the manuscript.

## ETHICS APPROVAL

All the preparation phases of the study were conducted in accordance with ethical and legal principles for data collection and statistical analysis. All clinical data were anonymized and analysed by an independent reviewer.

## Data Availability

Author elects to not share data.
